# Biological characteristics of a new long-chain fatty acid transport protein 1 from *Trichinella spiralis* and its participation in lipid metabolism, larval moulting, and development

**DOI:** 10.1186/s13567-024-01380-0

**Published:** 2024-09-30

**Authors:** Yang Li Li, Qi Qi Lu, Wen Wen Zheng, Zhao Yu Zhang, Jin Yi Wu, Mei Hao Wei, Xin Zhuo Zhang, Ruo Dan Liu, Zhong Quan Wang, Jing Cui

**Affiliations:** Department of Parasitology, School of Basic Medical Sciences, Zhengzhou University, Zhengzhou, 450052 China

**Keywords:** *Trichinella spiralis*, long-chain fatty acid transport protein 1 (TsFATP1), RNAi, lipid metabolism, moulting

## Abstract

Long-chain fatty acid transport protein 1 (FATP1) is a member of the fatty acid transporter family. It facilitates transmembrane transport of fatty acids and participates in lipid metabolism. Lipids are essential components of the cell and organelle membranes of *Trichinella spiralis.* The nematode has lost the capacity to synthesise the necessary lipids de novo and has instead evolved to obtain fatty acids and their derivatives from its host. This study aims to ascertain the primary biological characteristics and roles of *T. spiralis* FATP1 (TsFATP1) in lipid metabolism, larval moulting, and the development of this nematode. The results show that TsFATP1 is highly expressed at enteral *T. spiralis* stages, mainly localised at the cuticle, the stichosome and the intrauterine embryos of the parasite. The silencing of the *TsFATP1* gene by TsFATP1-specific dsRNA significantly decreases the expression levels of TsFATP1 in the worm. It reduces the contents of ATP, triglycerides, total cholesterol, and phospholipids both in vitro and in vivo. RNAi inhibits lipid metabolism, moulting, and the growth of this nematode. The results demonstrate that TsFATP1 plays an essential role in lipid metabolism, moulting, and the development of *T. spiralis.* It could also be a target candidate for the anti-*Trichinella* vaccine and drugs.

## Introduction

*Trichinella spiralis* is an intestine and tissue-dwelling nematode of the genus *Trichinella. Trichinella* infection results from ingesting raw or undercooked animal meat infected with *Trichinella* infectious muscle larvae (ML). It is widely distributed worldwide [1]. In China, from 2009 to 2020, eight outbreaks of human trichinellosis involving 479 cases and two deaths were reported [2]. Pork is the principal source of *Trichinella* infection, which is not only a serious public health problem but also a severe risk to meat food safety [3]. Therefore, it is necessary to develop an anti-*Trichinella* vaccine to interrupt the transmission of *Trichinella* infection within domestic food animals [4, 5].

When *T. spiralis*-infected meat is ingested, the ML are liberated from the capsule during gastric fluid digestion and are activated into intestinal infectious larvae (IIL) by bile and enteral contents. The IIL invade the enteral epithelium and moult four times to develop into adult worms (AW) in the gut’s epithelial intramulticellular niche [6, 7]. The AW deposits its newborn larvae (NBL), which invade skeletal muscle cells via the lymphatic and blood circulatory system; they are then encapsulated, ready to complete the life cycle [8, 9]. Moulting is an essential process for the growth and development of *T. spiralis,* and is a crucial function that allows it to adapt to the intestinal environment [10]. If the moulting process is impeded, the old epidermis is not entirely shed; as a result, the larvae will be wrapped in the moulting sheath and cannot develop into adulthood [11]. In addition, nutrients required for the parasite’s growth, development, and reproduction mainly come from the host. Furthermore, most parasites have a limited type of anabolic metabolism and lack the ability to synthesise de novo lipids [12, 13]. Therefore, the proteins involved in moulting and the lipid metabolism pathways of *T. spiralis* play crucial roles in its growth and development.

Long-chain fatty acid transport protein 1 (FATP1) belongs to the fatty acid transport proteins (FATPs) family. There are six members of the FATPs (FATP1-6), all with different tissue localisation and functions [13, 14]. Previous studies have shown that FATP1 promotes the transmembrane transport of fatty acids and participates in fatty acid metabolism [15]. Studies on the *FATP* gene of *Caenorhabditis elegans* showed that the *FATP* gene performs a vital function on the cuticle surface barrier [16], suggesting that the silencing of the *FATP* gene might impede the metabolism and development of the nematode [17]. However, there are, to date, no literature reports on the biological characteristics and functions of the *FATP* gene within *T. spiralis’* life cycle.

In the present study, the *T. spiralis FATP1* gene (*TsFATP1*; Genbank: KRY37099.1) was retrieved from the *T. spiralis* draft genome [18]. TsFATP1 was detected within *T. spiralis* circulatory proteins in murine serum samples at 2 and 8 weeks post-infection with liquid chromatography tandem mass spectrometry (LC–MS/MS) [19]. The FATP transports the lipids and might play a role in the nematode’s lipid metabolism and development, and in parasite-host interaction. This study aimed to ascertain the primary biological characteristics and roles of *TsFATP1* in *T. spiralis* lipid metabolism, larval moulting, and development.

## Materials and methods

### Parasite, cell, and experimental animals

*Trichinella spiralis* (ISS534) was collected from an infected domestic pig in central China and preserved and passaged in Kunming mice within our laboratory. The cell line used in the experiment was the human colon cancer epithelial cell (Caco-2 cell) bought from the Cell Bank of Chinese Academy of Sciences (Shanghai). Female BALB/c mice of 4-6-weeks-old were purchased from the Henan Provincial Experimental Animal Centre (Zhengzhou, China). All animal experiments were approved by the Life Science Ethics Committee of Zhengzhou University (No. ZZUIRB GZR 2021–0044).

### *T. spiralis* worm collection and protein preparation

Mice experimentally infected with 200 *T. spiralis* ML at 42 days post-infection (dpi) were sacrificed, and their murine skeletal muscles were digested using an artificial digestion method to collect the ML. Mice infected with 5000 ML were also euthanised at six hours post-infection (hpi), 3, and 6 days post-infection (dpi). The small intestine from each mouse was obtained, longitudinally dissected, and cut into segments 1 cm long. The intestinal segments were incubated in normal saline at 37 ℃ for 1 h. The IIL at 6 hpi, 3, and 6 dpi AW were collected as previously reported [20, 21]. The 6 dpi AW were washed nine times with phosphate-buffered saline (PBS) containing 100 μg/mL streptomycin and 100 U/mL penicillin and cultured in RPMI-1640 medium at 5% CO_2_ and 37 °C for 24 h. The culture solution was collected after filtration through a sterile sieve, and the NBL was obtained by centrifugation at 12 000 g for 30 min [22]. The worm somatic soluble and excretory/secretory (ES) proteins of different *T. spiralis* stages were prepared as described before [23]. In brief, *T. spiralis* worms at various stages were completely washed using sterile saline and cultured in RPMI-1640 medium (5000 worms/mL) in 5% CO_2_ at 37 °C for 18 h. After being centrifuged at 3000 × *g* for 20 min, the ES proteins in the supernatant were filtered using a 0.22 μm membrane and concentrated with an ultrafiltration tube. The concentration of the ES proteins was measured using the Coomassie brilliant blue G-250 method, and the ES proteins were stored at −80 °C before use [6].

### Bioinformatics analysis of TsFATP1

The full-length cDNA sequence of the *TsFATP1* gene was retrieved from GenBank (KRY37099.1). After searching the coding sequence and amino acid sequence of *TsFATP1*, the physicochemical properties and structure of *TsFATP1* were predicted and analysed using bioinformatics analysis tools (EXPASY, TMHMM, SignaIP, SMART, and Alphafold2) [24]. The prediction of structure, enzyme active sites and the substrate binding sites of *TsFATP1* was performed using the NCBI database and Pymol software [25].

### TsFATP1 sequence alignment and evolutionary tree construction

The FATP sequences of other species/genotypes of *Trichinella* and other nematodes were aligned by Clustal X and ESPript 3.0, and the phylogenetic tree was constructed by MEGA11 software and the Neighbor-joining (NJ) method [26]. The *Trichinella* species/genotypes and other organisms used in this study were *T. spiralis* (KRY37099.1), *T. nativa* (KRZ53724.1), *T. murrelli* (KRX47772.1), *T. patagoniensis* (KRY11832.1), *T. nelsoni* (KRX7163.1), *Trichinella* T6 (KRY37100.1), *Trichinella* T9 (KRX65023.1), *T. papuae* (KRZ68392.1), *T. zimbabwensis* (KRZ14626.1), *T. pseudospiralis* (KRX89808.1), *Trichuris trichiura* (CDW54271.1), *Caenorhabditis elegans* (NC_003284.9), *Homo sapiens* (NP_940982.1), and *Mus musculus* (NP_001344110.1).

### Expression of TsFATP1 and preparation of anti-rTsFATP1 serum

Total RNAs were isolated from *T. spiralis* ML using Trizol reagent (Invitrogen, USA) and were reversely transcribed into the cDNA. The complete *TsFATP1* cDNA sequence was amplified using PCR by specific primers with *BamH* I and *Hind* III restriction enzyme sites **(bold**). The specific primers were 5'-AC**GGATCC**ATGCTTCAGATGCGTCATGGGTTG-3' and 5'-CCC**AAGCTT**TTATATATTCATTCGCCCTTC-3'. The PCR products were cloned into the expression vector pQE-80L with a His-tag at N-terminus (Novagen, USA), and recombinant pQE-80L/TsFATP1 was introduced into *E. coli* BL21 (Novagen) [23]. The expression of rTsFATP1 was induced at 16 °C for 3 days using 1.5 mM isopropyl β-d-1-thiogalactopyranoside (IPTG) [27]. rTsFATP1 was purified by nickel column affinity chromatography (Sangon Biotech, Shanghai, China). Expression of the rTsFATP1 protein was analysed and identified by SDS-PAGE and western blotting [28].

Twenty female BALB/c mice aged 4–6 weeks were first immunised with 20 μg of rTsFATP1, fully emulsified with Freund’s complete adjuvant [29]. The mice were injected subcutaneously with 20 μg emulsified rTsFATP1 four times, once every 14 days. The Freund’s complete adjuvant was replaced with Freund’s incomplete adjuvant for the remaining immunisations. Seven days after the last immunisation, tail blood was taken from the immunised mice, serum samples were isolated, and ELISA measured the titre of anti-rTsFATP1 IgG with rTsFATP1 as a coating antigen [4].

### SDS-PAGE and western blot analysis

Soluble proteins of diverse *T. spiralis* worm phases (ML, IIL, 3 and 6 d AW, and NBL) and rTsFATP1 were separated by 10% SDS-PAGE at 80 V for 30 min and then 120 V for 75 min [24]. The proteins were transferred onto a polyvinylidene fluoride membrane (PVDF) (Millipore, USA) in a semi-dry transfer cell (Bio-Rad, USA) [30]. The membrane was blocked with 5% skimmed milk in Tris-buffered saline containing 0.05% Tween (TBST) at 37 °C for 1 h and cut into strips. The strips were probed at 37 °C for 2 h by diverse sera (1:100 dilutions of anti-rTsFATP1 serum, *T. spiralis*-infected mouse sera, and normal mouse serum) and 1:1000 dilutions of anti-His-tag monoclonal antibody (McAb). After being washed with TBST, the strips were incubated at 37 °C for 1 h with HRP-anti-mouse IgG conjugate (1:10 000; Sangon Biotech). After further washes, the colour was developed with either 3,3'-diaminobenzidine tetrahydrochloride (DAB; Sigma-Aldrich) or an enhanced chemiluminescent kit (FeiYuBIO, NanTong, China) [20, 31].

### qPCR assay of TsFATP1 expression levels at various *T. spiralis* stages

Total RNAs of diverse *T. spiralis* stage (ML, IIL, 3 and 6 d AW, and NBL) were extracted using Trizol reagent (Invitrogen). The expression level of TsFATP1 mRNA at various stages was assessed using qPCR, as described previously [7]. The TsFATP1-specific primers for qPCR analysis were 5'-CTTCAGATGCGTCATGGGTT-3' and 5'-GCTCAATGTAACACCAAACG-3'. The relative expression level of TsFATP1 mRNA was normalised by subtracting the mRNA expression level of a *T. spiralis* housekeeping gene *GAPDH* (GenBank: AF452239) [32, 33] and then calculated using the comparative Ct ^2−ΔΔCt^ method [34]. Each test had three replicates.

### Indirect immunofluorescent assay (IIFA)

IIFA was performed to localise the worm tissue location of TsFATP1 in various *T. spiralis* stages, as reported previously [35, 36]. The fresh whole worms of diverse *T. spiralis* stages (ML, 6 and 12 h IIL, 3 and 6 d AW, and NBL) were collected and fixed with 4% paraformaldehyde. After being washed and rehydrated in various concentrations of alcohol, the worms were embedded in paraffin and 2-µm thick cross-sections were cut with a microtome [37]. The cross-sections were deparaffinised in xylene and rehydrated. The whole, intact worms and cross-sections were blocked with 5% goat serum at 37 °C for 2 h. After three washes in PBST, they were incubated overnight at 4 ℃ with various sera (1: 10 dilutions of anti-rTsFATP1 serum, *T. spiralis*-infected mouse sera as positive control sera, and normal mouse sera as negative control sera). After being washed again, they were incubated with Alexa Fluor 488 conjugated anti-mouse IgG (1:100; Sangon Biotech.). Then, the complete larvae and AW and their cross-sections were observed under fluorescence microscopy (Olympus, Japan) [21, 27].

### RNA interference (RNAi)

On the basis of the full-length cDNA sequence of TsFATP1, three pairs of TsFATP1-specific primers containing T7 promoter (underlined) and enhancer (**bold**) were designed (5'-**GATCACTAATACGACTCACTATAGGG**AGGTTGAATCTGGCCAGACGTG-3', 5'-**GATCACTAATACGACTCACTATAGGG**GCGTAATTGTTGCGGCTTTC-3'; 5'-**GATCAC** TAATACGACTCACTATAGGGGAACGAGGTTTGTGCATTCGATG-3', 5'-**GATCACTAATACGACTCACTATAGGG**AGGATTAAAACCCTCTTTGACC-3'; 5'-**GATCACTAATACGACTCACTATAGGG**ATGCAAGGTATTTCCTGATGG-3', 5'-**GATCACTAATACGACTCACTATAGGG**CCTCGTTCATCA CGCATCAAT-3'). Green fluorescent protein (GFP) was used as a negative control, and its primers were as follows: 5'-**GATCAC** TAATACGACTCACTATAGGGTCCTGGTCGAGCTGGACGG-3', 5'-**GATCACTAATACGACTCACTATAGGG**CGCTTCTCGTTGGGGTCTTTG-3' [38]. The primers were synthesised and prepared by Sangon Biotech (Shanghai, China). The larval survival rate from each group was observed, and the optimal type, concentration and specificity of the dsRNA, as well as the culture time, were ascertained [39]. The transcription levels of the *TsFATP1 *gene in the ML after silencing the *TsFATP1* gene were assessed by qPCR, as described previously [40]. Furthermore, ML crude soluble proteins were prepared, and the TsFATP1 protein expression level of dsRNA-transfected and control ML was assessed using western blotting analysis. The GAPDH protein expression level was also analysed as an internal gene control. Moreover, to verify the specificity of dsRNA-TsFATP1, the mRNA and protein expression levels of a *T. spiralis* type C lectin (TsCTL, GenBank: KRY42391.1) in the dsRNA-treated larvae were also ascertained using qPCR and Western blot [41].

### Effect of TsFATP1 gene silencing on larval lipid metabolism

To observe the effect of *TsFATP1* gene silencing on larval lipid metabolism, 3000 ML were transfected with 50 ng/μL of dsRNA-FATP1 using electroporation methods [38]. Briefly, 3000 ML were suspended in 100 μL of electroporation buffer containing 50 ng/μL of dsRNA-FATP1. The larva suspension was electroporated (200 V, 200 Ω, 25 µF) using a Gene Pulser II System (Bio-Rad, USA), then added to an RPMI 1640 culture medium of up to 500 μL and incubated at 37 °C and 5% CO_2_ for 2 days. The contents of ATP as well as the triglyceride and cholesteryl esters of different groups of treated ML were measured using an ATP kit (Sangon Biotech) [42], triglyceride kit (Beyotime Biotech) [17], and cholesterol kit (Nanjing Jiancheng Biological Engineering) [43], respectively. The lipid was extracted from different groups of the ML, and the phosphorus content in the lipid was measured using the tissue inorganic phosphorus content detection kit (Sangon Biotech) to determine the changes in phospholipid content within the different groups [44]. In addition, oil red O staining was used to detect the distribution of lipid droplets in the whole intact ML, and ImageJ software (NIH Image, Bethesda, MD, USA) was used to quantify the lipid droplets in the midgut of three groups of larvae [45].

### The in vitro larval invasion and moulting test

To determine the role of TsFATP1 in *T. spiralis* larval invasion of the intestinal epithelia, the in vitro larval invasion test was carried out as reported before [46, 47]. In brief, the transfected ML were first incubated with 5% pig bile for 2 h at 37 °C and activated into the IIL. Caco-2 cell monolayers were overlaid with 50 larvae suspended in a semi-solid medium (DMEM + 1.75% agarose). After being cultured at 37 °C for 2 h, larval intrusion into the monolayer was observed under microscopy. The larvae that had entered the monolayer and migrated within it were assessed as invasive larvae, whereas the larvae that still existed on the surface of the Caco-2 monolayer and exhibited the spiral coil were assessed as non-invasive [48]. Additionally, larval moulting was also observed and counted under a light microscope after being cultured for 2 days [8, 41]. Each test had triplicates.

### Challenge infection of mice with dsRNA-TsFATP1 transfected ML

One hundred and twenty female 6-week-old mice were divided into dsRNA-TsFATP1, GFP, and PBS groups (40 mice per group). Each group of mice was orally infected with 200 ML transfected by 50 ng/μL dsRNA-TsFATP1. Ten mice of each group were sacrificed at 12 hpi, 3 dpi, 6 dpi, and 35 dpi, and the IIL, AW and ML were collected and counted [49, 50]. The worm reduction of intestinal IIL and AW, and the muscle larval burden (larvae per gram of muscles, LPG) were evaluated according to worm burdens of the TsFATP1-dsRNA group compared to the PBS group. Ten females from each group were cultivated, and the female adult fecundity (reproductive capacity) was assessed based on the number of NBL produced by each female in 72 h [27]. The lengths of 30 worms from various groups of infected mice were observed and measured under microscopy. The expression of native TsFATP1, the contents of ATP, triglyceride, cholesteryl esters, and the phospholipid levels in different-stage worms from various groups were measured as before. Finally, to observe the inhibitory role of RNAi on *T. spiralis* growth and development, the moulting of fifty IIL worms from three groups of infected mice was also observed and numbered [37].

### Statistical analysis

GraphPad Prism 10.0 was used to plot and analyse the data, and the data in this study were shown as the mean ± standard deviation (SD). One-way ANOVA was used to analyse the difference in relative expression of TsFATP1 mRNA and protein, the content of ATP, triglyceride, total cholesterol and phospholipid, worm burdens and length. The differences in larval invasion and moulting rate among various groups were analysed using the Chi-square test. The statistical difference level was *P* < 0.05.

## Results

### Bioinformatics analysis of TsFATP1

**Figure 1:**
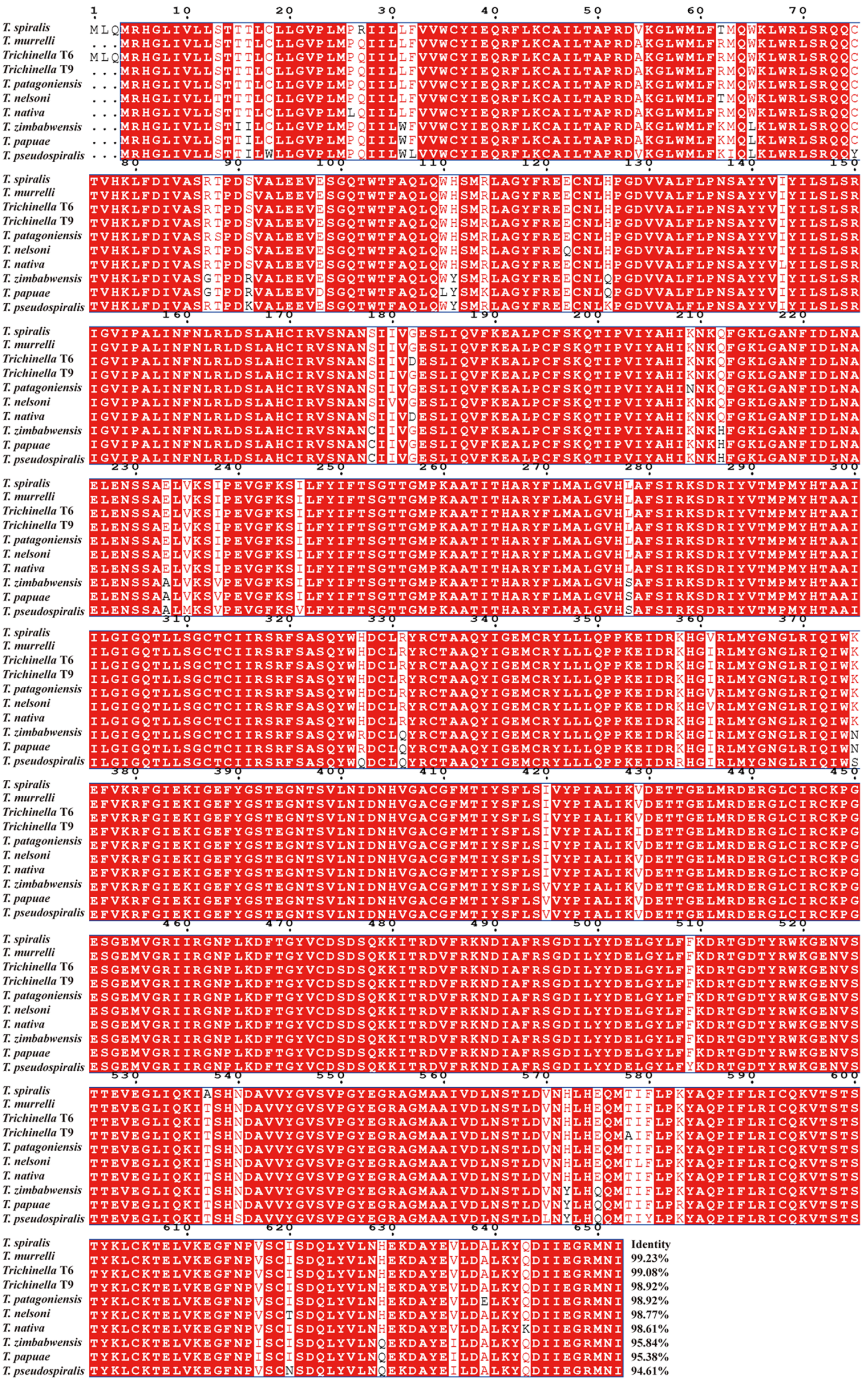
**Multi-sequence alignment of TsFATP1 with FATP of different species or genotypes of the genus** ***Trichinella*.** According to the analysis of Clustal X and ESPript 3.0, the backgrounds of the same amino acids are marked in dark red, and the conservative substitution of amino acid residues are marked in light red. The *FATP* genes have high homology. The number at the end of each sequence represents the percentage of identity with TsFATP1.

Bioinformatics analysis showed that the complete TsFATP1 cDNA sequence was 1959 bp, encoding was 653 aa, and the molecular weight was 70.169 kDa with pI 8.54. The TsFATP1 homology was compared with those of other *Trichinella* species or genotypes, and the results are shown in Figure 1. The TsFATP1 amino acid sequence had an identity of 99.23, 99.08, 98.92, 98.92, 98.77, 98.61, 95.84, 95.38, and 94.61% of FATP sequences of other various *Trichinella* species/genotypes (*T. murrelli*, *Trichinella* T6, *Trichinella* T9, *T. patagoniensis*, *T. nelsoni*, *T. nativa*, *T. zimbabwensis*, *T. papuae*, and *T. pseudospiralis*).

**Figure 2:**
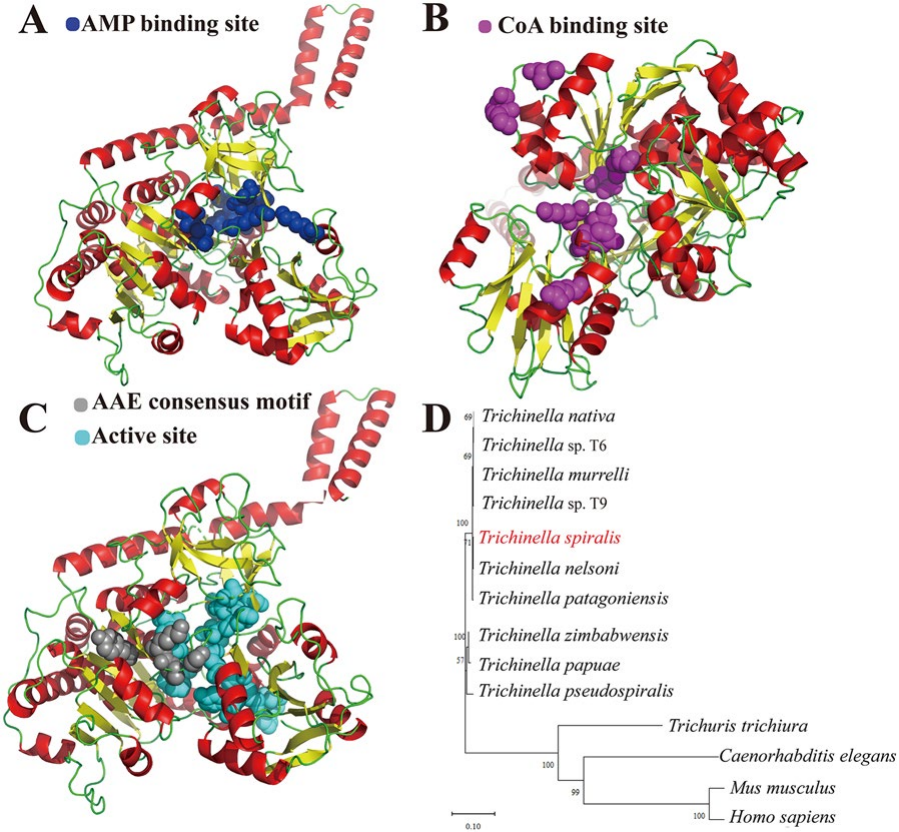
**Tertiary structure prediction and evolutionary tree construction of TsFATP1. A** The adenosine AMP binding sites of TsFATP1 were localised at Thr-253, Asn-368, Gly-369, Glu-389, Thr-394, Asp-498, Phe-510, Arg-513, and Lys-604. **B** CoA binding sites of TsFATP1 were localised at Met-293, Pro-352, Pro-353, Ile-356, Asn-368, Lys-521, Glu-523, Glu-576, and Tyr-585. **C** The active sites of TsFATP1 were localised at Thr-253, Met-293, Pro-294, leu-350, Pro-352, Pro-353, Ile-356, Asn-368, Glu-389, Thr-394, Asp-498, Phe-510, Arg-513, Lys-521, Asn-524, and Tyr-585, and the common motifs were localised at Tyr-250, Thr-253, Gly-258, Pro-260, and Lys-261. **D** The evolutionary tree of TsFATP1 was inferred using the NJ method. The bootstrap consensus tree inferred from 1000 replicates represents the evolutionary history of the taxa analysed. The encapsulated and non-encapsulated species of the genus *Trichinella* were localised in two different evolutionary clades of *Trichinella*.

The N-terminal of TsFATP1 has a typical hydrophobic structure. Five typical transmembrane helical regions were localised among amino acids 7–29, 141–163, 246–268, 295–317, and 406–428. There were twelve B-cell epitopes with an AFD class I domain. Alphafold2 predicted the tertiary structure of TsFATP1, and the results were visualised using Pymol. We can see the binding sites of adenosine monophosphate (AMP) (Figure 2A) and coenzyme A (CoA) (Figure 2B) and the active sites and acyl activase (AAE) common motifs in the domain (Figure 2C). The phylogenetic tree analysis showed a monophyletic group of the genus *Trichinella*. Within the genus *Trichinella*, two clear clades were revealed: one was the clade of seven encapsulated species/genotypes (*T. spiralis*, *T. nelsoni*, *T. patagoniensis*, *Trichinella* T6, *T. nativa*, *T. murrelli*, and *Trichinella* T9), and the other was the clade of three non-encapsulated species (*T. zimbabwensis*, *T. papuae*, and *T. pseudospiralis*) (Figure 2D).

### Expression and antigenic identification of rTsFATP1

**Figure 3:**
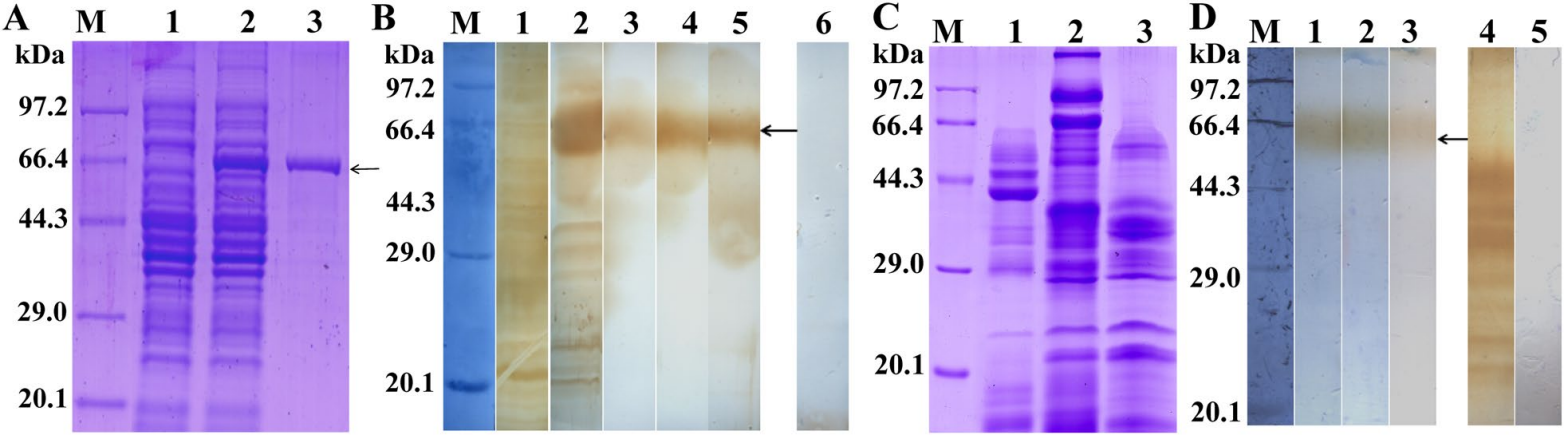
**Expression and identification of rTsFATP1. A** SDS-PAGE analysis of rTsFATP1. Lane M: Protein marker. Lane 1: lysate of recombinant *E. coli* incorporating pQE-80L/TsFATP1 prior to induction. Lane 2: lysate of recombinant *E. coli* incorporating pQE-80L/TsFATP1 post-induction. Lane 3: purified rTsFATP1.** B** Western blot analysis of rTsFATP1 antigenicity. Lane 1: Lysates of pQE-80L/TsFATP1 before induction were not recognised by infection serum. Lane 2: Lysates of pQE-80L/TsFATP1 post-induction were recognised by infection serum. The purified rTsFATP1 was recognised by infection serum (lane 3), anti-rTsFATP1 serum (lane 4), and anti-his tag McAb (Lane 5) (black arrow), but not by normal murine serum (lane 6). **C** SDS-PAGE analysis of ES proteins of various *T. spiralis* stages. Lane 1–3: ES proteins of ML, IIL and 6 d AW. **D** Western blotting analysis of ES proteins of various *T. spiralis* stages. Lane 1–3: ES proteins of ML, IIL and 6 d AW were identified by anti-rTsFATP1 serum (black arrow). The ML ES proteins were recognised by infection serum (lane 4), but not by normal serum (lane 5).

The results of SDS-PAGE analysis showed that the BL21 bacteria carrying pQE-80L/ TsFATP1 expressed a 66.4 kDa fusion protein band. After being purified with Ni–NTA Sefinose Column, the rTsFATP1 protein showed a clear single band (Figure 3A). The molecular weight (66.4 kDa) of the rTsFATP1 was the same as its predicted size. To assess the antibody response induced by rTsFATP1 immunisation, the titer of anti-rTsFATP1 IgG at 7 d after the fourth immunization was assayed by ELISA. The results showed that the IgG titer of anti-rTsFATP1 immune serum reached 1:10^4^, indicating that rTsFATP1 has good antigenicity. Using western blot analysis, the purified rTsFATP1 was identified by anti-rTsFATP1 serum, infection serum and anti-his tag monoclonal antibodies but not by normal murine serum (Figure 3B). Additionally, ES proteins of ML, IIL and 6 d AW were identified by anti-rTsFATP1 serum, suggesting that TsFATP1 was a secretory protein of this nematode (Figures 3C, D).

### Expression level of TsFATP1 in different *T. spiralis* stages

**Figure 4:**
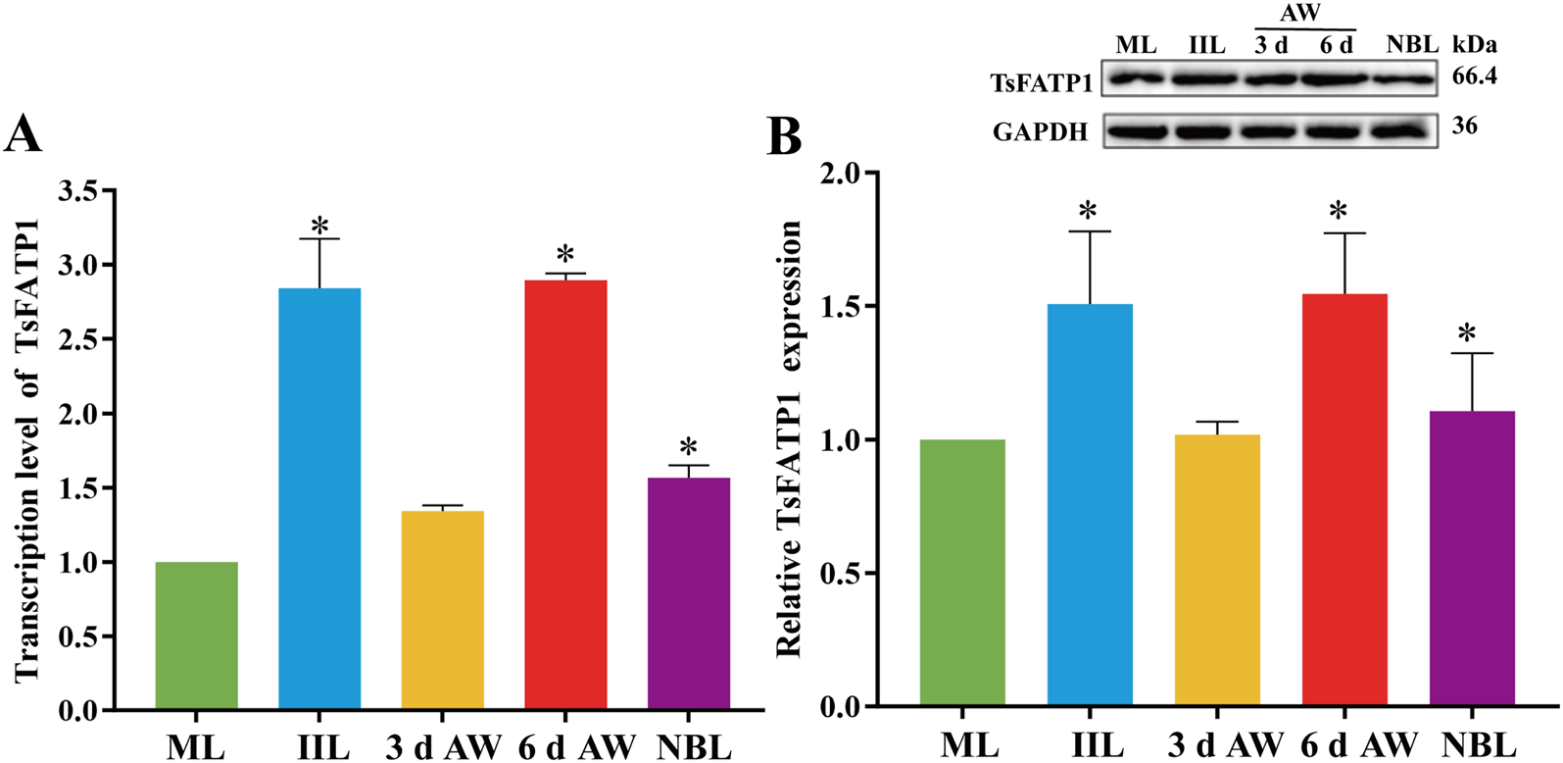
**Transcription and expression of TsFATP1 in different** ***T. spiralis*** **stages. A** qPCR analysis of TsFATP1 transcription levels in different *T. spiralis* stages.** B** Western blot analysis of the TsFATP1 expression level in somatic soluble proteins of different *T. spiralis* stages. *indicates a significant difference in TsFATP1 transcription and expression levels compared to the ML stage (*P* < 0.01).

The qPCR results showed that the transcription level of TsFATP1 at various *T. spiralis* stages was significantly different (*F* = 95.17, *P* < 0.0001). The transcription level of TsFATP1 at the IIL, 6 d AW, and NBL stages was obviously higher than that at the ML stage (*t*_IIL_ = 9.550, *t*_6d AW_ = 71.24, *t*_NBL_ = 11.50, *P* < 0.001) (Figure 4A). Western blot results showed that the difference in TsFATP1 expression levels in various worm stages was also statistically significant (*F* = 6.160, *P* < 0.01). The TsFATP1 expression level at the IIL, 6 d AW, and NBL stages was also evidently higher than that at the ML stage (*t*_IIL_ = 3.210, *t*_6 d AW_ = 4.145, *t*_NBL_ = 0.8709, *P* < 0.05) (Figure 4B).

### Expression and worm localisation of native TsFATP1 at various *T. spiralis* stages

**Figure 5:**
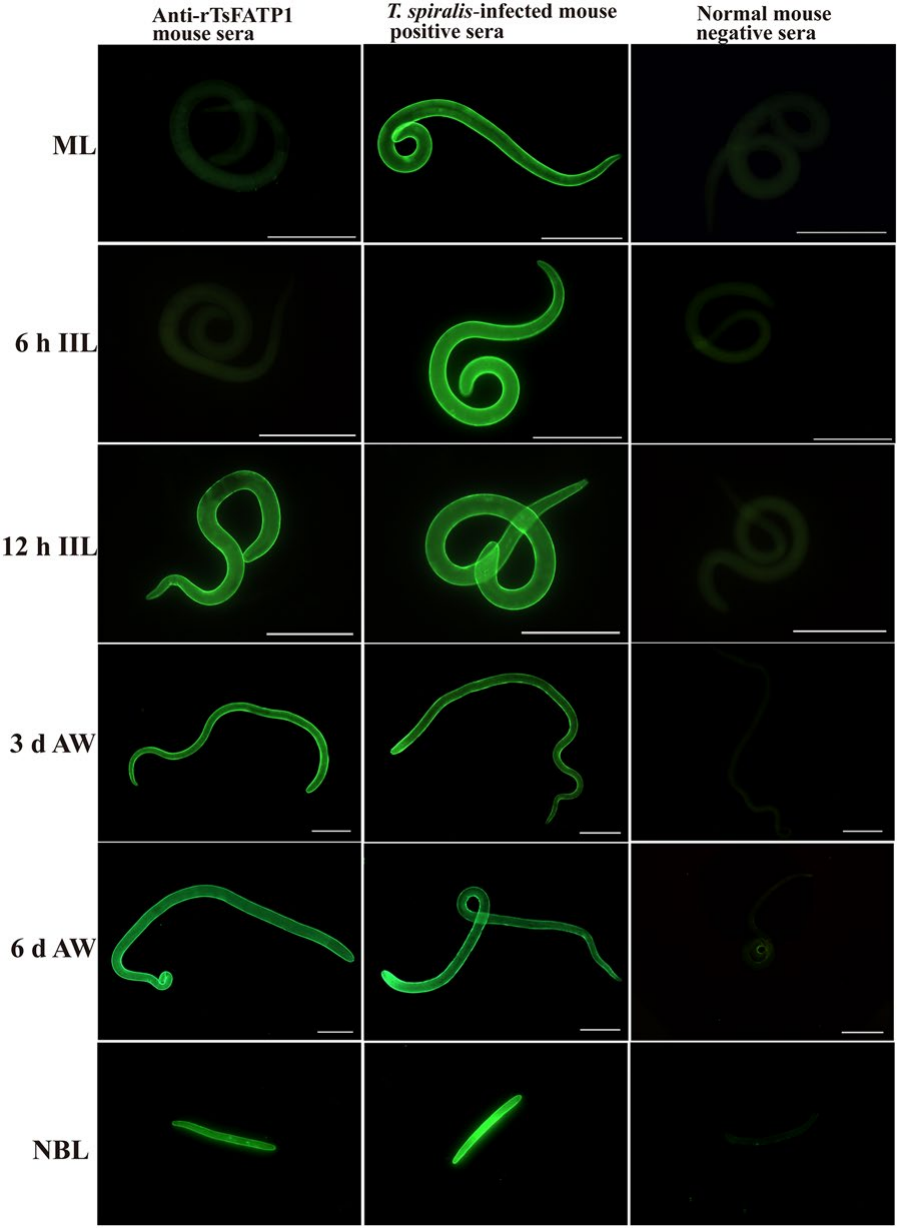
**Expression of TsFATP1 at the cuticle of various *T. spiralis*** **stages by IIFA.** The whole intact worms were probed by anti-rTsFATP1 serum, and immune fluorescence staining was observed at the epicuticle of 12 h IIL, 3 d and 6 d AW, and NBL. However, normal murine serum did not recognise any worm components of the nematode. Scale bars of ML, IIL and AW: 200 μm; NBL scale bars: 100 μm.

**Figure 6:**
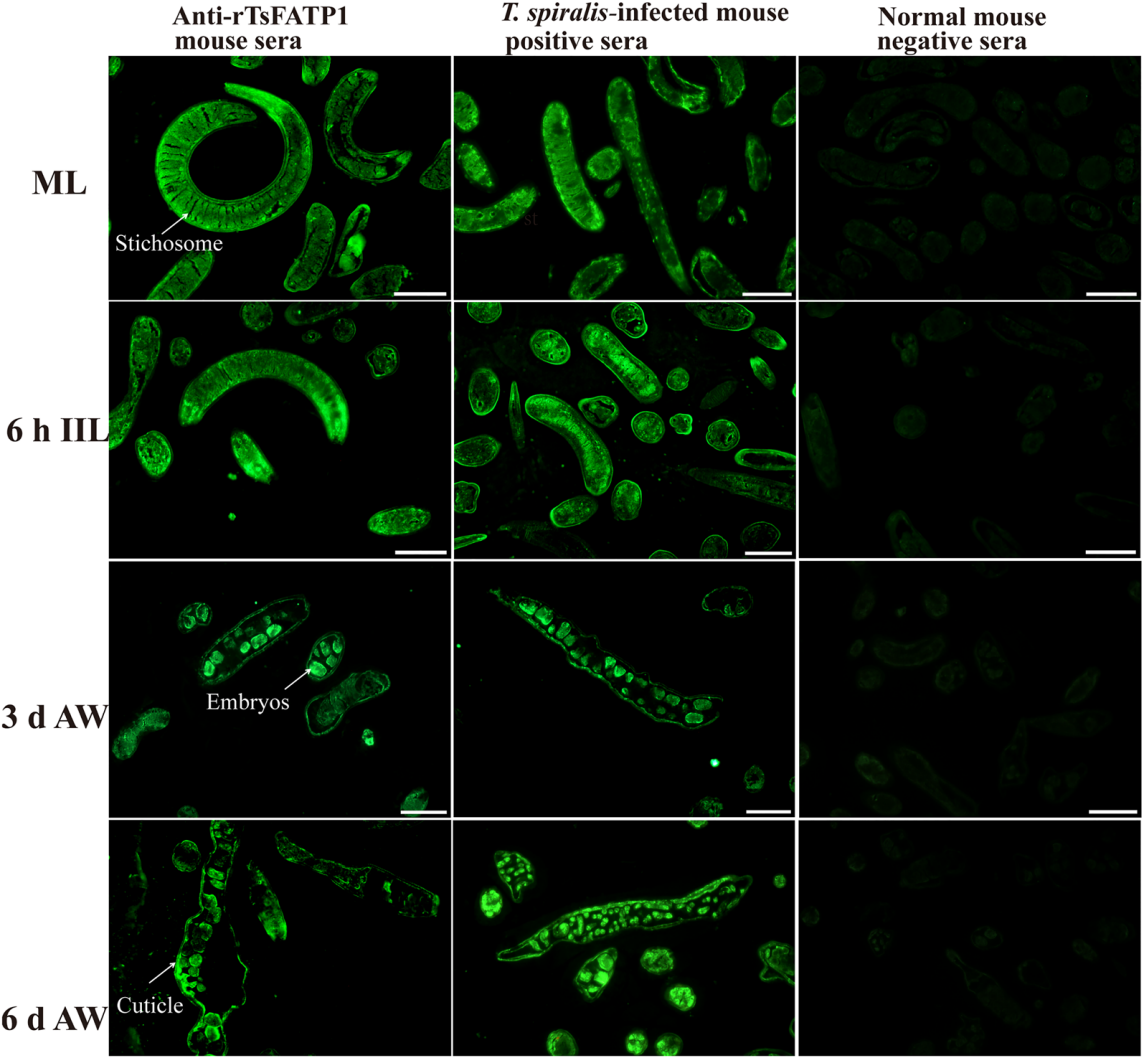
**Immunolocalization of TsFATP1 in worm cross-sections of diverse** ***T. spiralis*** **stages by IIFA.** Green fluorescence staining was observed at the cuticle, the stichosome and the female intrauterine embryos. No immunostaining in worm cross-sections was observed using normal serum as a negative control. Scale bars: 100 μm.

The results of IIFA within whole worms revealed that bright green fluorescence was observed on the external cuticle of 12 h IIL, 3 and 6 d AW, and NBL by anti-TsFATP1 serum, but not at ML and 6 h IIL stages. This indicates that TsFATP1 was expressed at 12 h IIL, AW, and NBL, but not at the ML and early IIL (Figure 5) stages. The results of IIFA within worm cross-sections showed that immunostaining was primarily localised within cuticle, stichosome and female adult worm’s intrauterine embryos (Figure 6).

### Reduction of TsFATP1 expression after silencing the TsFATP1 gene

**Figure 7:**
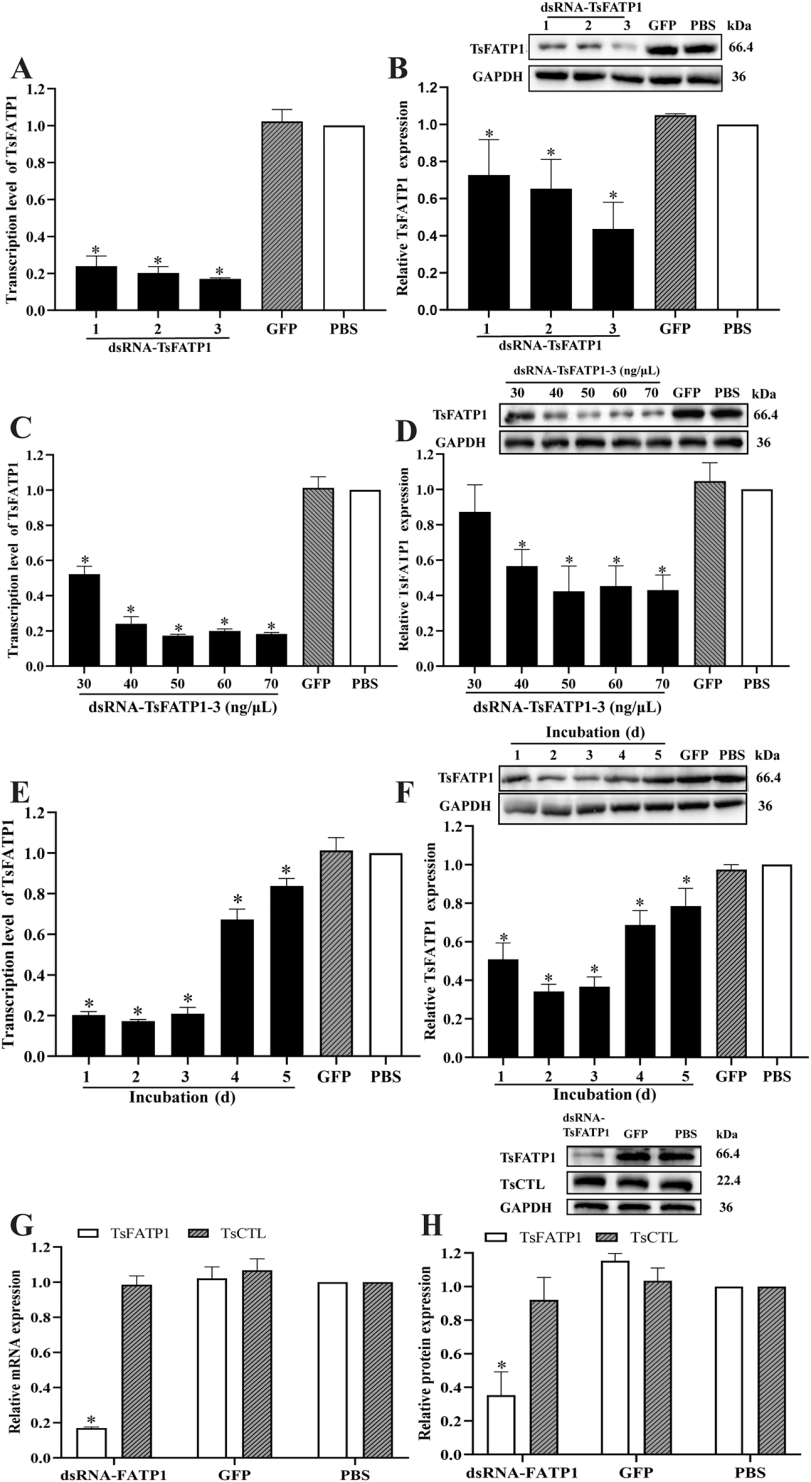
**Silencing TsFATP1 gene suppressed TsFATP1 expression A** TsFATP1 transcription levels in ML transfected with three kinds of dsRNA-FATP1. **B** TsFATP1 expression levels in ML transfected with three kinds of dsRNA-FATP1. **C** TsFATP1 transcription levels in ML transfected with various doses of dsRNA-FATP1-3.** D** TsFATP1 expression levels in ML transfected with various doses of dsRNA-FATP1-3. **E** TsFATP1 transcription levels in ML at 1–5 d after transfection with 50 ng/μL dsRNA-FATP1-3. **F** TsFATP1 expression levels in ML at 1–5 d after transfection with 50 ng/μL dsRNA-FATP1-3. **G** Transcriptional levels of TsFATP1 and TsCTL in ML treated by dsRNA-FATP1. **H** Expression levels of TsFATP1 and TsCTL in ML treated by dsRNA-FATP1. *P < 0.05 relative to the PBS group.

The ML were divided into five groups (dsRNA-FATP1-1–3, dsRNA-GFP, and PBS groups) and then cultured for 3 days after electroporation. The results showed that the larval survival rate of dsRNA-TsFATP1-1, 2, 3, dsRNA-GFP, and PBS was 91.62, 91.76, 91.85, 91.74, and 91.77%, respectively (*F* = 0.4095, *P* > 0.05), indicating that electroporation had no distinct effect on larval survival rate. Compared with the PBS group, the transcriptional levels of TsFATP1-1–3 were inhibited by 76.02, 79.75, and 83.01%, respectively (*F* = 349.7, *P* < 0.0001) (Figure 7A). Furthermore, expression levels of TsFATP1 protein were inhibited by 27.37, 34.70, and 56.40%, respectively (*F* = 11.78, *P* < 0.001) (Figure 7B), demonstrating that dsRNA-TsFATP1-3 was more effectively suppressive. Different concentrations of dsRNA-TsFATP1-3 (30, 40, 50, 60, and 70 ng/μL) were used, and the TsFATP1-silenced ML were cultured for 2 days. Transcription levels of the *TsFATP1* gene were inhibited by 47.73, 75.85, 82.74, 80.02, and 81.64%, respectively (*F* = 390.0, *P* < 0.0001) (Figure 7C), and TsFATP1 protein expression was inhibited by 12.71, 43.36, 57.70, 54.64, and 57.02%, respectively (*F* = 458.0, *P* < 0.0001) (Figure 7D). When 50 ng/μL dsRNA-TsFATP1 were used, and the ML were cultured for different times (1–5 d), the transcription levels of the *TsFATP1* gene were inhibited by 79.70, 82.74, 79.18, 32.65, and 16.2%, respectively (*F* = 334.0, *P* < 0.0001) (Figure 7E), and the expression of TsFATP1 protein was inhibited by 49.18, 65.88, 63.38, 31.36, and 21.44%, respectively (*F* = 59.60, *P* < 0.0001) (Figure 7F). This indicates that both transcription and protein expression levels of the *TsFATP1* gene decreased significantly 2 days after culture. In the ML transfected with dsRNA-TsFATP1, the mRNA and protein expression levels of a *T. spiralis* type C lectin (TsCTL) had not obviously reduced (*P* > 0.05) (Figure 7G, H), indicating that dsRNA-TsFATP1 had a specific silencing effect on the *TsFATP1* gene.

### Suppression of dsRNA on larval lipid metabolism

**Figure 8:**
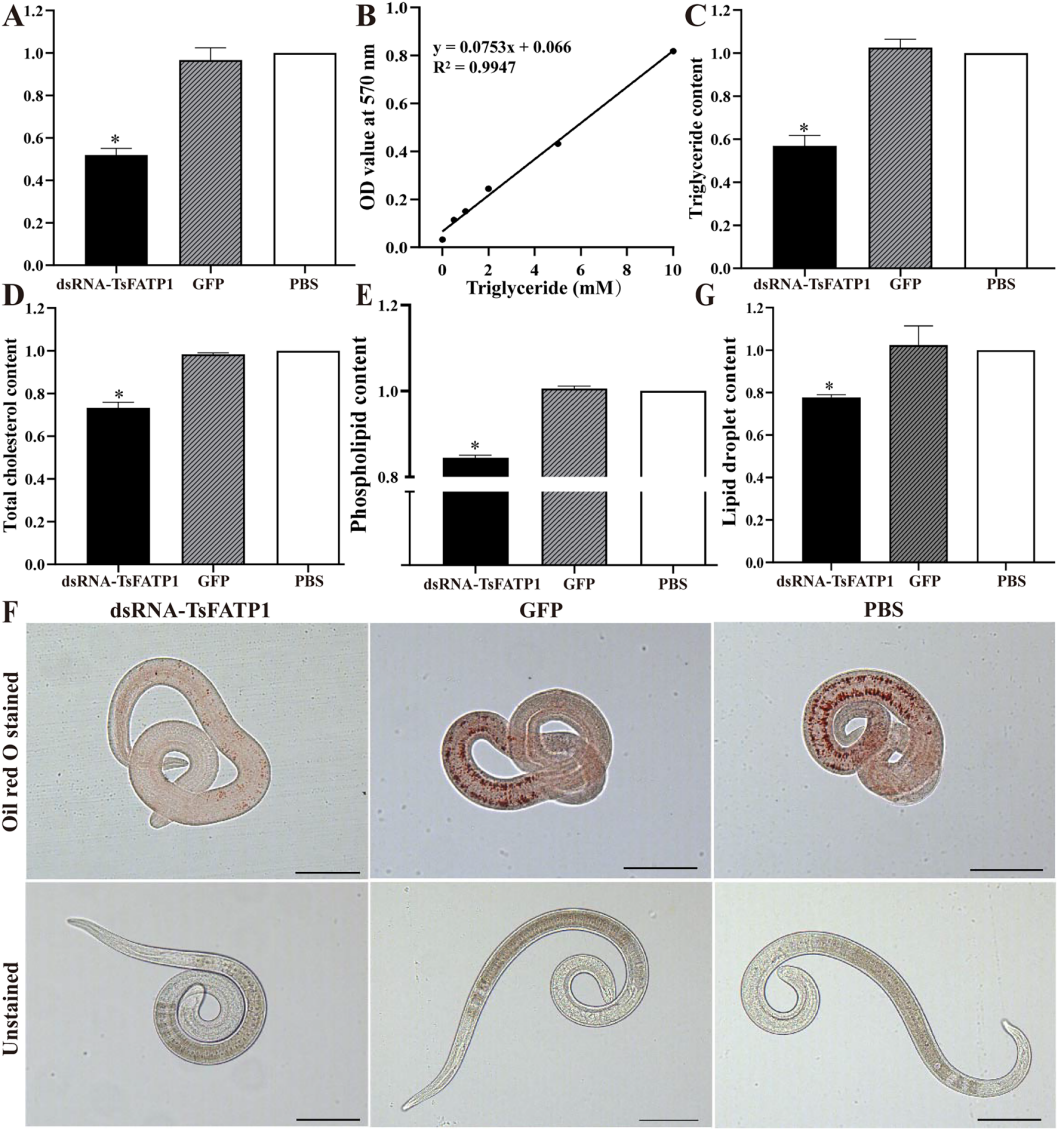
**Suppression of dsRNA on lipid metabolism of *T. spiralis*** **ML. A** dsRNA reduced ATP content in ML. **B** triglyceride standard curve. **C** dsRNA reduced larval triglyceride content.** D** dsRNA reduced total larval cholesterol content. **E** dsRNA decreased larval phospholipid content. **F** distribution of lipid droplets in three groups of the ML. **G** dsRNA decreased larval lipid droplets. *indicates *P* < 0.05 compared to the PBS group. Scale bars: 200 μm.

The silencing of the *TsFATP1* gene inhibited larval lipid metabolism. After RNAi, the ATP contents of dsRNA-TsFATP1, dsRNA-GFP, and PBS groups were 2.6187 × 10^–2^, 4.882 × 10^–2^, and 5.0514 × 10^–2^ μmol, respectively. Compared with the PBS group, the ATP content of dsRNA-TsFATP1 group decreased by 40.15% (*F* = 149.2, *P* < 0.0001) (Figure 8A). A triglyceride standard curve was drawn and is shown in Figure 8B. The triglyceride content of the three groups was 8.47, 4.89, and 14.88 mM, respectively, and the triglyceride content in the dsRNA-TsFATP1 group decreased by 43.13%, compared to the PBS group (*F* = 153.9, *P* < 0.0001) (Figure 8C). The total cholesterol content of the three groups was 0.339, 0.4555, and 0.4633 mmol, and the total cholesterol content of the dsRNA-TsFATP1 group was reduced by 26.81% (*F* = 258.3, *P* < 0.0001) (Figure 8D). The content of phosphorus in the ML lipid of the three groups was 8.1 × 10^–2^, 9.64 × 10^–2^, and 9.59 × 10^–2^ mmol, respectively, and the phospholipid content of the dsRNA-TsFATP1 group decreased by 15.55% (*F* = 1012,* P* < 0.0001) (Figure 8E). Oil red O staining showed that small lipid droplets were distributed throughout the muscle larvae, mainly within the intestine and tail. The red colour of treated larvae faded after RNAi (Figure 8F), and the content of lipid droplets in the dsRNA-TsFATP1 group reduced by 22.23% compared with the PBS group (*F* = 19.78, *P* = 0.0023) (Figure 8G).

### dsRNA reduced *T. spiralis* larval invasion and ecdysis in Caco-2 monolayer

**Figure 9:**
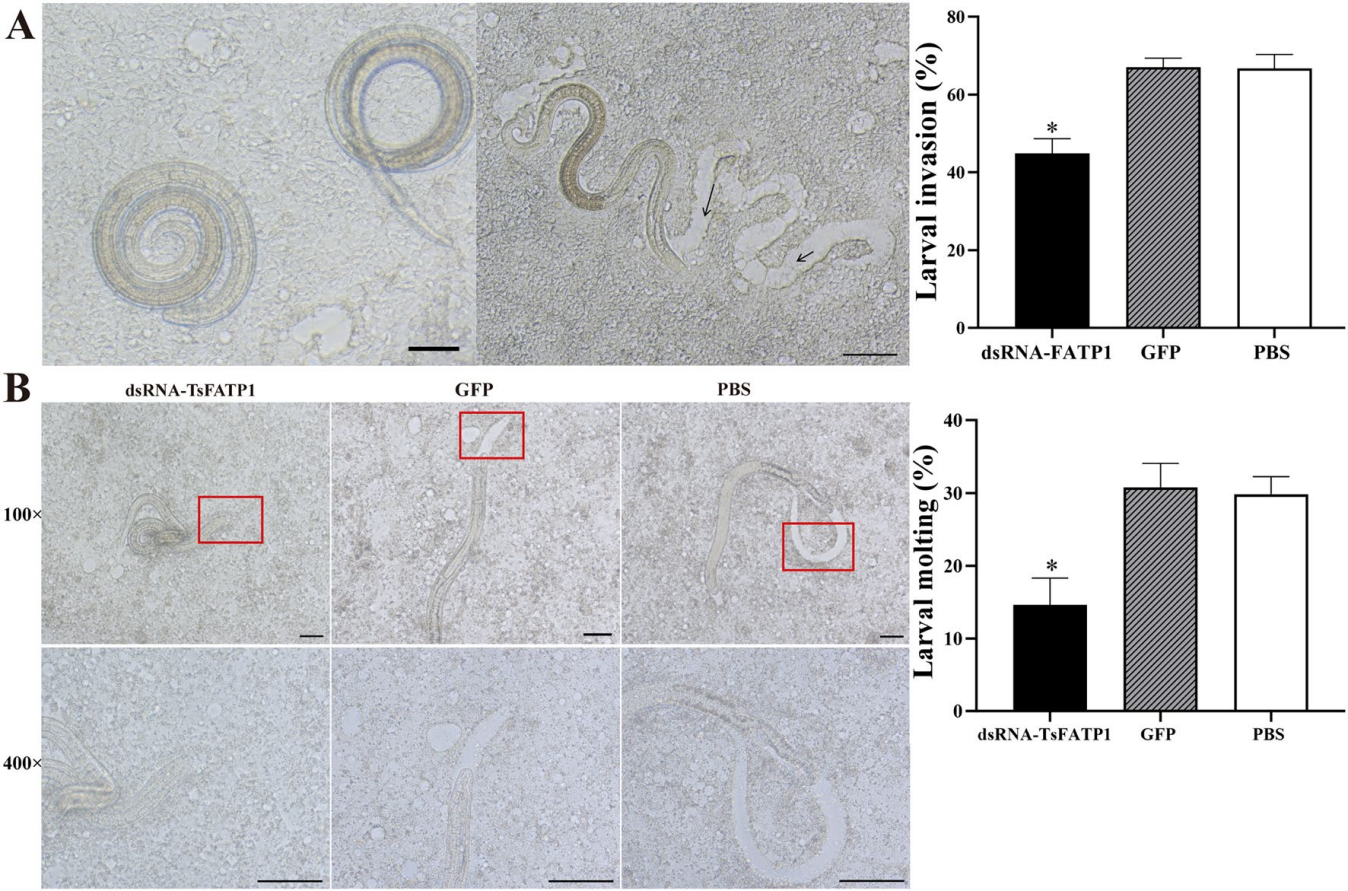
**Inhibition of dsRNA on the in vitro *T. spiralis*** **invasion and moulting. A** larval invasion into Caco-2 monolayers after RNAi. **Left:** The non-invaded larvae were spirally coiled on the surface of Caco-2 cells. Scale bar: 100 μm. **Right:** invaded larvae were locomotive and migratory, and the integrity of the larvae-invaded Caco-2 monolayer was destroyed; the migrating trace was visible and is marked with black arrows. Scale bars: 50 μm. **B** larval moulting of three groups after RNAi. Larval moulting was clearly suppressed by dsRNA; no obvious sheath at the larval tail of the dsRNA-TsFATP1 group was observed, whereas clear moulting sheathes were seen in the GFP and PBS control groularvalps. Scale bars: 200 μm. * indicates *P* < 0.05 compared with the PBS group.

After the transfected and activated *T. spiralis* larvae were added to the Caco-2 monolayer and incubated for 2 h, the larval invaded the monolayer and migrated (Figure 9A). The larval invasion rates in dsRNA-TsFATP1, GFP, and PBS groups were 44.84, 67.05, and 66.67%, respectively. Compared with the PBS group, larval invasion inhibition of the dsRNA-TsFATP1 group was 32.74% (*χ*^*2*^ = 13.971, *P* < 0.01), suggesting that silencing the *TsFATP1* gene significantly inhibited the in vitro larval invasion of Caco-2 monolayer. After the larvae were incubated for 2 days and examined, the larval moulting rates in the dsRNA-TsFATP1, GFP, and PBS groups were 14.63, 30.78, and 29.86%, respectively. Compared with the PBS group, the larval moulting of the dsRNA-TsFATP1 group was inhibited by 51% (*χ*^*2*^ = 4.656,* P* < 0.05) (Figure 9B), indicating that larval moulting and development were distinctly inhibited by dsRNA-TsFATP1, and suggesting that TsFATP1 participated in larval moulting and the development of the life cycle of *T. spiralis*.

### Inhibition of RNAi on the in vivo larval development and moulting

**Figure 10:**
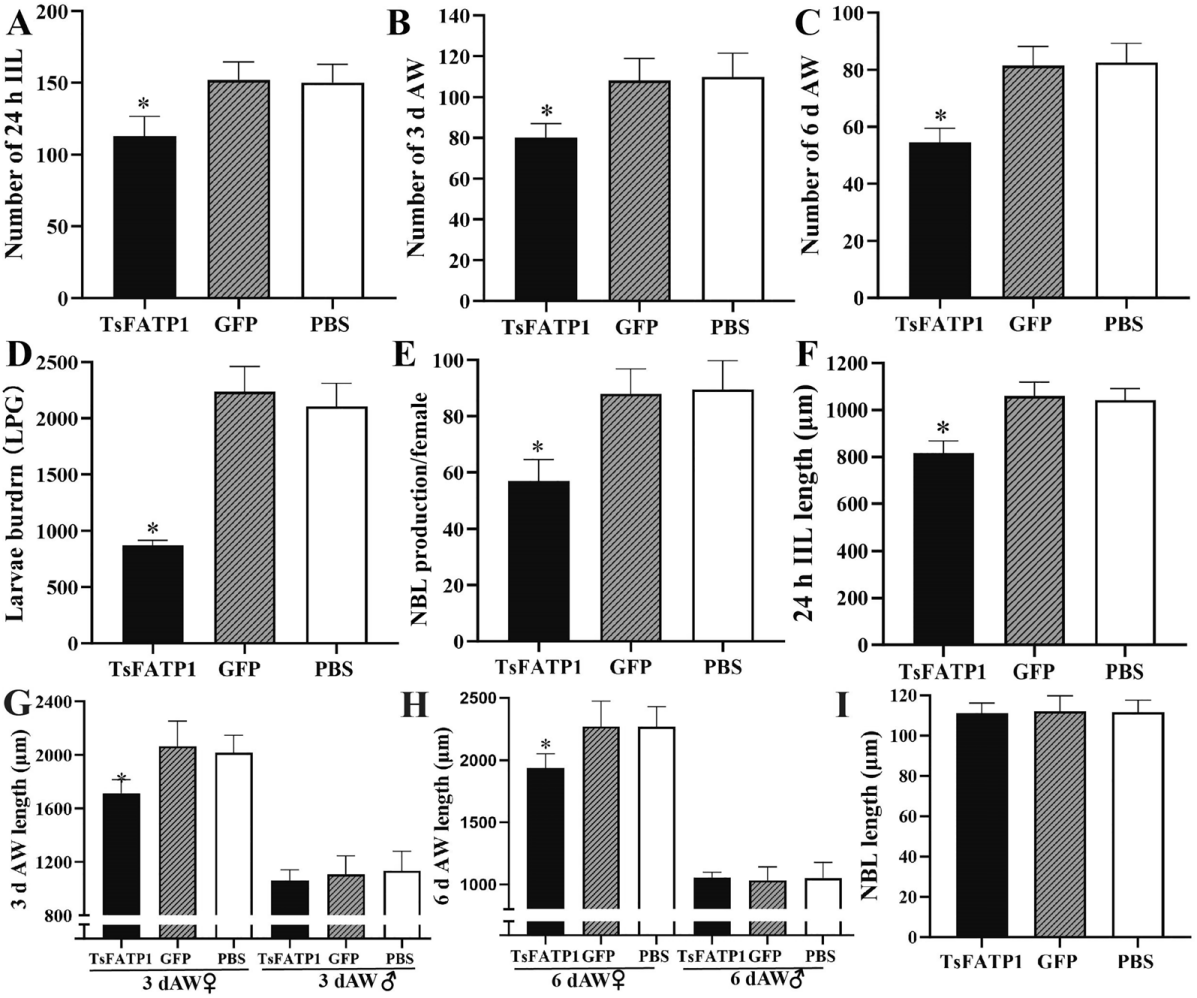
**Inhibition of RNAi on in vivo larval invasion and development in infected mice. A** the number of 24 h IIL. **B** the number of 3 d AW. **C** the number of 6 d AW. **D** muscle larval burden at 35 dpi.** E** NBL production/female. **F** the length of 24 h IIL. **G** the length of 3 d AW. **H** the length of 6 d AW.** I** the length of NBL. *indicates *P* < 0.0001 compared with PBS group.

The results of animal challenge experiments showed that compared to the PBS group, the worm burdens of 24 h IIL, 3 and 6 d AW, and ML of the dsRNA-TsFATP1 group were reduced by 24.97, 27.05, 33.94, and 58.6%, respectively (*F*_24 h IIL_ = 29.25, *F*_3 d AW_ = 27.95, *F*_6 d AW_ = 65.29, *F*_ML_ = 178.6, *P* < 0.0001) (Figures 10A–D). The NBL production in 72 h of the dsRNA-TsFATP1 group reduced by 36.31% (*F* = 41.69, *P* < 0.0001) (Figure 10E). Additionally, the length of 24 h IIL, 3 and 6 d female adults from the dsRNA-TsFATP1 group decreased by 21.80, 15.08, and 14.48% (*F*_IIL_ = 206.1, *F*_3 d female_ = 51.34, *F*_6 d female_ = 40.44, *P* < 0.0001), respectively (Figures 10F–H). However, there was no significant difference in the length of male adults and NBL from various other groups (*P* > 0.05) (Figures 10H, I).

### dsRNA-TsFATP1 impeded larval moulting in infected mice

**Figure 11:**
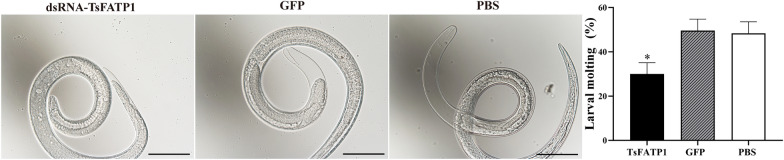
**Inhibition of dsRNA-TsFATP1 on the in vivo *T. spiralis*** **larval moulting in infected mice.** *P < 0.001 compared with the PBS group. Scale bars: 200 μm.

The moulting of 24 h IIL in dsRNA-TsFATP1, GFP, and PBS group was 30, 48, and 49.33%, respectively (Figure 11). Compared with the PBS group, the inhibition rate of larval moulting in the dsRNA-TsFATP1 group was 39.19% (*χ*^*2*^ = 17.761,* P* < 0.001), suggesting that TsFATP1 is involved in IIL moulting during larval development.

### dsRNA-TsFATP1 inhibited TsFATP1 expression in IIL and adults from infected mice

**Figure 12:**
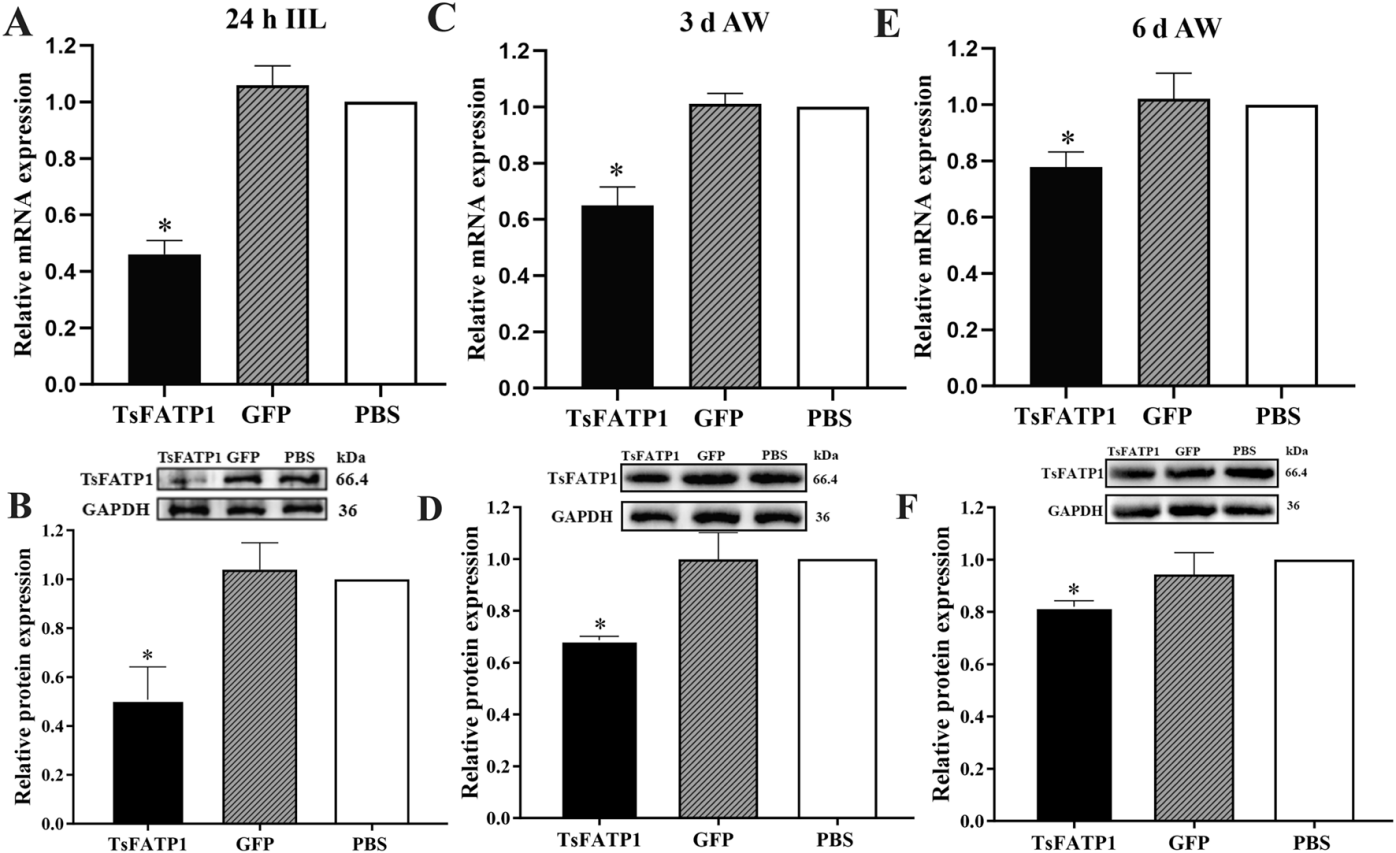
**RNAi suppressed transcription and expression of TsFATP1 in IIL and adults from infected mice**. **A**, **C** and **E** dsRNAi-TsFATP1 inhibited TsFATP1 transcriptional level in 24 h IIL (**A**), 3 d AW (**C**) and 6 d AW (**E**). **B**, **D** and **F** dsRNAi-TsFATP1 inhibited TsFATP1 expression level in 24 h IIL (**B**), 3 d AW (**D**) and 6 d AW (**F**). *indicates *P* < 0.05 compared with the PBS group.

The transcription and expression levels of TsFATP1 in IIL, 3 and 6 d AW after RNAi were assayed by qPCR and western blot. The results showed that transcription and expression levels of TsFATP1 in the dsRNA-TsFATP1 group were significantly lower than those in the PBS group, and its transcription levels were inhibited by 53.92, 35.01, and 22.24%, respectively (*F*_IIL_ = 140.4, *F*_3 d AW_ = 67.57, *F*_6 d AW_ = 14.57, *P* < 0.0001) (Figures 12A–C). Its expression levels were suppressed by 49.31, 31.29, and 18.01%, respectively (*F*_IIL_ = 26.02, *F*_3 d AW_ = 26.87, *F*_6 d AW_ = 10.22, *P* < 0.05) (Figures 12D–F).

### dsRNAi-TsFATP1 inhibited lipid metabolism in IIL and adults

**Figure 13:**
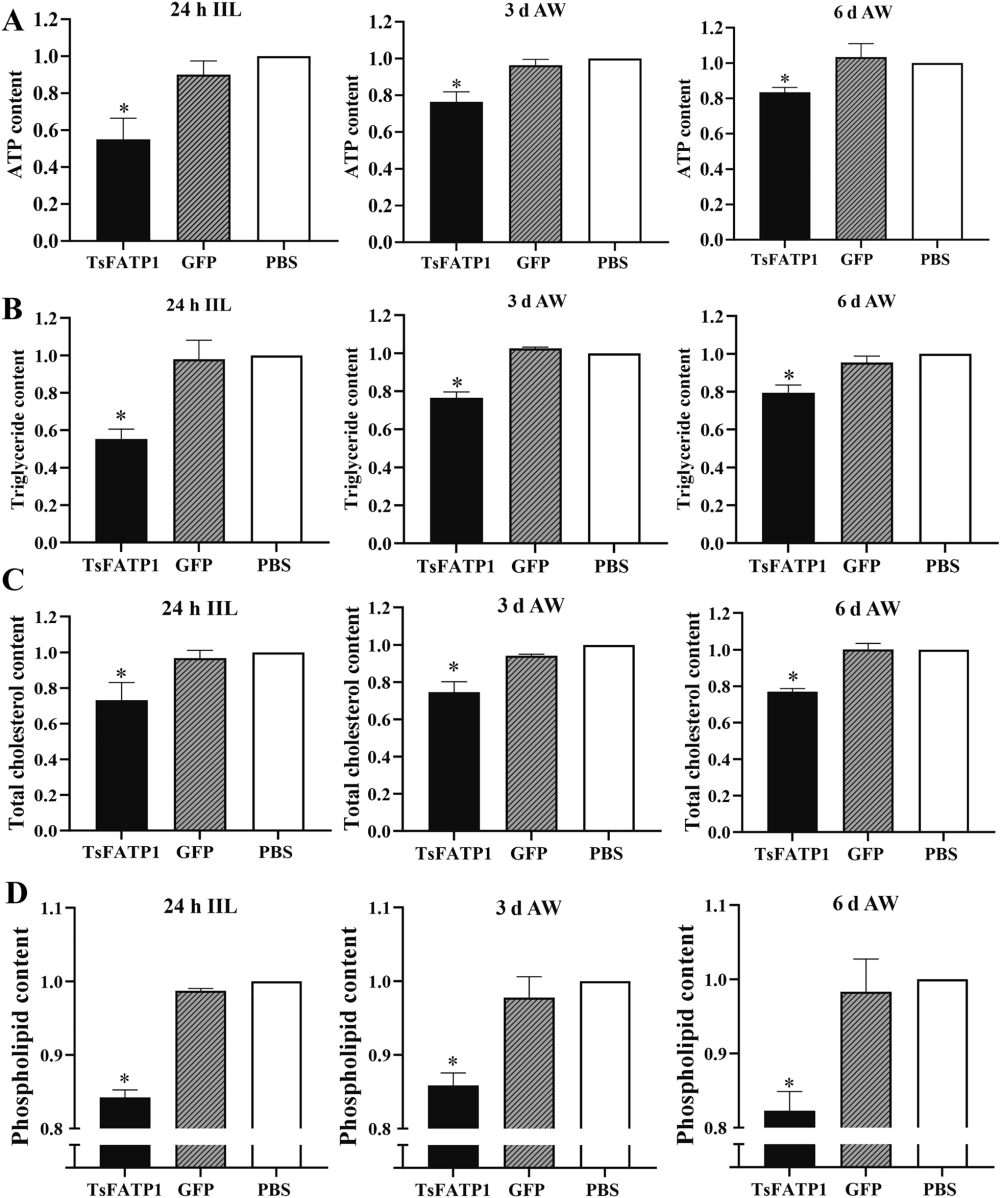
**dsRNA-TsFATP1 reduced the contents of ATP (A), triglyceride (B), cholesterol (C) and phospholipid (D) in 24 h IIL, 3 and 6 d AW**. *indicates *P* < 0.01 compared with the PBS group.

Compared to the PBS group, the ATP content of the IIL, 3 and 6 d AW dsRNA-TsFATP1 groups decreased by 45, 23.64, and 16.67%, respectively (*F*_IIL_ = 26.80, *P* < 0.01; *F*_3 d AW_ = 36.75, *P* < 0.001; *F*_6 d AW_ = 15.50, *P* < 0.01) (Figure 13). In the dsRNA-TsFATP1 group, the triglyceride content in the three-stage worms had decreased by 44.64, 23.36, and 20.53%, respectively (*F*_IIL_ = 43.90, *F*_3 d AW_ = 181.3, *F*_6 d AW_ = 37.55, *P* < 0.001). The total cholesterol content in the three-stage worms had decreased by 26.75, 25.52, and 22.95% (*F*_IIL_ = 16.19, *F*_3 d AW_ = 47.75, *F*_6 d AW_ = 119.9, *P* < 0.01), and the phospholipid content had reduced by 15.78, 14.14, and 17.71%, respectively (*F*_IIL_ = 588.7, *F*_3 d AW_ = 48.92, *F*_6 d AW_ = 33.43,* P* < 0.001).

**Figure 14:**
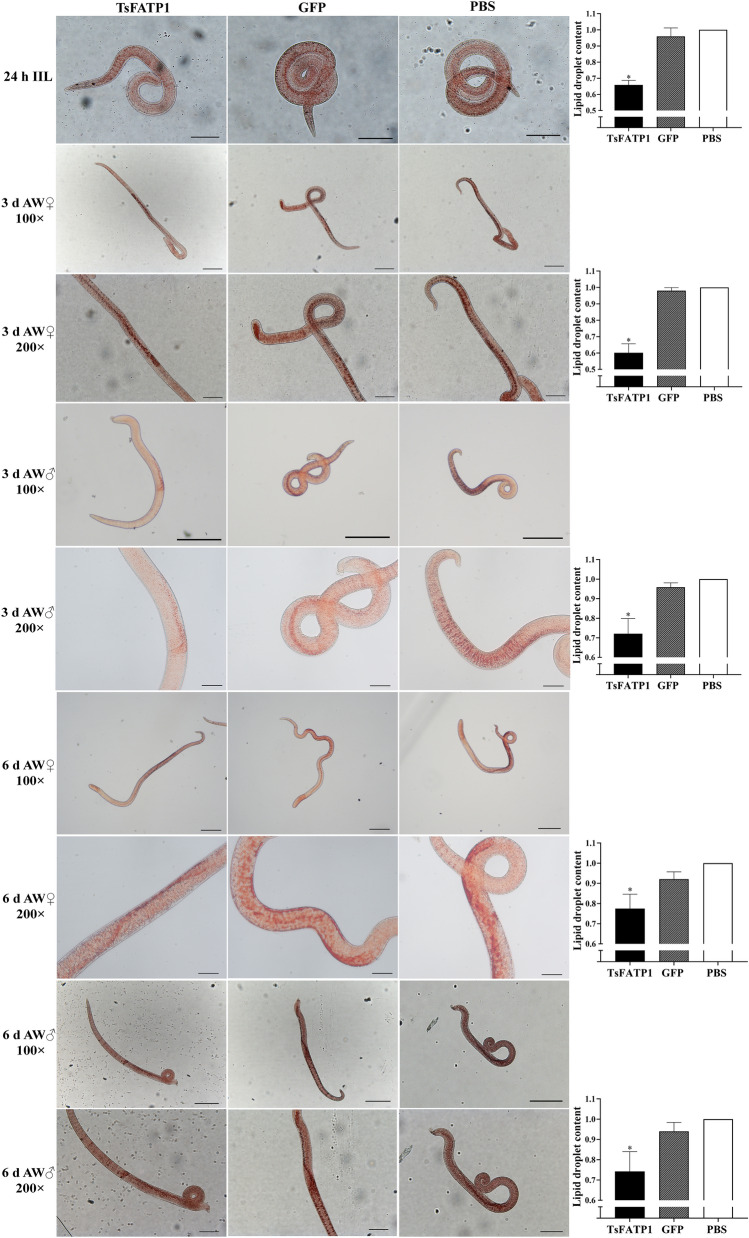
**dsRNA-TsFATP1 reduced lipid droplet contents in IIL and AW from infected mice.** The 24 h IIL scale bars: 100 μm; 3 and 6 d AW 100 × scale bars: 200 μm; 3 and 6 d AW 200 × scale bars: 100 μm. * indicates *P* < 0.01 compared with the PBS group.

Moreover, the distribution of lipid droplets was observed using oil red O staining, and the results showed that tiny lipid droplets were distributed throughout the worm but were principally localised in the intestine and tail, the ovary of adult females, and the testis of adult males (Figure 14). Image analysis showed that compared to the PBS group, the lipid droplet content in IIL, 3, and 6 d females and males of the dsRNA-TsFATP group had reduced by 36.16, 39.85, 27.92, 22.5, and 25.81% (*F*_IIL_ = 116.8, *F*_3 d female_ = 133.5, *F*_3 d male_ = 31.42, *F*_6 d female_ = 18.61, *F*_6 d male_ = 14.21, *P* < 0.01).

## Discussion

Currently, albendazole is the drug of choice for the treatment of trichinellosis [51]. However, due to the complex life cycle of *Trichinella* spp., albendazole is effective against intestinal *Trichinella* stages but less effective in killing the encapsulated ML in the muscle stage [52, 53]. Therefore, therapeutic interventions for trichinellosis remain limited. The search for alternative, less toxic, and safer agents remains an ongoing and challenging effort. Lipids are molecules related to various biological processes and are essential for all life forms. Fatty acids are necessary for collagen synthesis, cuticle formation, and nematode larval development [54]. Lipids are important components of the cell and organelle membranes of *Trichinella* adults and larvae. Lipids include phospholipids, monoglycerides, free fatty acids, sterols, diglycerides, triglycerides, and sterol esters. They also act as signalling molecules in the intracellular information transmission of parasites [55]. The nematode has lost the capacity to synthesise the necessary lipids de novo and has thus evolved the ability to obtain fatty acids and their derivatives from its host [56]. The nematode-specific FATP family helps to facilitate lipid acquisition. It also promotes the transportation of fatty acids across the cell membrane [57]. Therefore, the FATP family proteins involved in the lipid metabolic pathway of *Trichinella* worms might be the ideal targets for the anti-*Trichinella* vaccine and therapeutic drugs.

In this study, the coding sequence of the *TsFATP1* gene was 1959 bp, encoding 653 aa with a molecular weight of 66.4 kDa and pI 8.54. *TsFATP1* had a hydrophobic structure at the N-terminus, five transmembrane structures, and no signal peptide. It had an AFD class I domain containing an adenosine monophosphate (AMP) binding site, a coenzyme A (CoA) binding site, an active site, and a common acyl activase (AAE) motif. Multiple sequence alignment with other species/genotypes of the genus *Trichinella* revealed that the TsFATP1 investigated in this study had high homology with several other encapsulated species of *Trichinella*. The phylogenetic tree constructed by integrating FATP sequences of various *Trichinella* species also showed that TsFATP1 had a closer evolutionary relationship to other encapsulated *Trichinella* species, indicating that the amino acid sequence of TsFATP1 is conserved and highly homologous. The full-length *TsFATP1* gene was cloned into a pQE-80L plasmid and expressed in the *E. coli* expression system. As the His-tag had only six histidines, rTsFATP1 purification was convenient and scarcely affected rTsFATP1, so an individual rTsFATP1 protein was acquired following purification with a Ni–NTA column [58].

After purification, the TsFATP1 was immunogenic and produced anti-TsFATP1 immune serum. Mice were immunised with TsFATP1, which triggered a high level of specific anti-TsFATP1 IgG response. The anti-rTsFATP1 IgG antibody titer reached 1:10^4^ 1 week following the last immunisation, suggesting that rTsFATP1 had a good antigenicity. Western blot results revealed that rTsFATP1 was recognised by anti-rTsFATP1 serum, infection serum, and anti-his tag McAb but not by normal murine serum. The transcription and expression levels of the *TsFATP1* gene in different *T. spiralis* developmental stages were investigated using qPCR and western blot. The results showed that the *TsFATP1* gene was transcribed and expressed in all stages of *T. spiralis*, and the transcription and expression levels of TsFATP1 were higher in IIL and 6 d AW stages than in the other stages (ML and NBL). The IIFA with worm cross-sections showed that TsFATP1 was mainly localised in the cuticle, the stichosome, and the embryos of the female adult worms. This finding suggested that the *TsFATP1* gene may be involved in the essential physiological activities of the various intestinal *T. spiralis* stages [49]. Moreover, the natural TsFATP1 in somatic crude proteins and ES proteins of IIL, ML, and AW was identified by anti-rTsFATP1 serum, demonstrating that TsFATP1 was a worm somatic and secretory protein. These findings suggested that TsFATP1 as a surface and excretory/secretory protein was directly in contact with the host intestinal epithelium, and it might participate in the larval invasion of gut mucosa in the early stage of *Trichinella* infection [21, 28].

RNA interference technology has been widely used to study the gene function of various parasites [59, 60]. In this study, the function of the *TsFATP1* gene in *T. spiralis* lipid metabolism and larval development was analysed by RNAi. After the ML were treated with 50 ng/μL dsRNA-TsFATP1 and cultured for 2 d, the mRNA and protein expression levels of *TsFATP1* gene had prominently decreased, and the contents of ATP, triglycerides, total cholesterol, and phospholipids were significantly reduced, indicating that RNAi inhibited larval lipid metabolism. As a result, the synthesis of ATP and lipid metabolites was also reduced [61]. The high expression of TsFATP1 in the intestinal *T. spiralis* phase suggested that TsFATP1 might be involved in the larval invasion of intestinal epithelium [30, 62]. The results of subsequent in vitro invasion tests and animal challenge experiments revealed that TsFATP1-specific dsRNA distinctly inhibited larval invasion of the gut mucosa.

A cuticle covers the nematode worm body, and moulting is the most remarkable feature and key step necessary for the growth and development of the nematode. If larvae ecdysis is impeded, the nematode will not develop properly [63]. Moulting is also a crucial strategy that intestinal nematodes use to adapt to the gut environment [64]. In this study, in vitro and in vivo larval moulting was significantly suppressed after the *TsFATP1* gene was silenced by RNAi. In addition, the worm burden of 24 h IIL, 3, and 6 d AW, and ML, and female adult fecundity evidently decreased. The length of IIL and AW, as well as their content of ATP, triglycerides, cholesterol, and phospholipids, had also reduced. Similarly, the RNAi silencing of the *Caenorhabditis elegans* fatty acid-binding protein 6 gene (*Ce-far-6* gene) significantly decreased the worm’s body length [17]. The results demonstrated that TsFATP1 plays a vital role in lipid metabolism, growth, and the development of intestinal *T. spiralis* stages. Additionally, the transcription and expression levels of the *TsFATP1* gene in the ML gradually began to increase 4–5 days after RNAi. RNAi did little to suppress the lipid metabolism of next-generation NBL and ML. This suggests that the knockdown of the *TsFATP1* gene in the ML stage causes a lipid metabolic disorder of ML, as well as the intestinal *T. spiralis* stage (IIL and AW). It also impedes larval moulting and development and has no obvious effect on the next generation of NBL and ML [8]. Previous studies showed that *Caenorhabditis elegans* with mutant acs-20 was the homologous to mammalian FATP and impaired cuticle structural integrity, suggesting that FATP is required for cuticle surface barrier function against small molecule permeability [16].

In conclusion, in this study, a novel *TsFATP1* gene was cloned, expressed, and characterised. Its biological properties and function within lipid metabolism, larval moulting and development of *T. spiralis* were assessed. The results showed that TsFATP1 was highly expressed at intestinal *T. spiralis* stages (e.g., IIL and AW stages), mainly localised at the cuticle, the stichosome, and around the embryos of female adults. TsFATP1 had good immunogenicity. The silencing of the *TsFATP1* gene by TsFATP1-specific dsRNA distinctly reduced the transcription and expression levels of TsFATP1 in the larvae, and the contents of ATP, triglycerides, total cholesterol, and phospholipids were significantly decreased in vitro and in vivo. The results demonstrated that TsFATP1 participated in lipid metabolism, larval moulting, and the development of *T. spiralis*. It could be considered a target candidate for vaccines and therapeutic drugs against larval moulting and nematode development.

## Abbreviations

AW: adult worms
DAB: 3, 3'-Diaminobenzidine tetrahydrochloride
ES: excretory/secretory (ES)
FATP1: long-chain fatty acid transport protein 1
GFP: green fluorescent protein
HRP: Horseradish peroxidase
IIFA: indirect immunofluorescent assay
IIL: intestinal infectious larvae
IPTG: isopropyl β-d-1-thiogalactopyranoside
LPG: larvae per gram of muscles
McAb: monoclonal antibody
ML: muscle larvae
NBL: newborn larvae
NJ: neighbor-joining
PBS: phosphate-buffered saline
PVDF: polyvinylidene fluoride membrane
TBST: Tris-bufered saline containing Tween
TsCTL: *T. spiralis* Type C lectin
TsFATP1: *T. spiralis* FATP1
